# Halitosis and Its Association with Sleep Quality and Psychological Factors Among University Students: A Cross-Sectional Study

**DOI:** 10.3390/ijerph23081043

**Published:** 2026-08-11

**Authors:** Juliana Maria Altavista Sagretti Gallo, Amanda Rafaelly Honório Mandetta, Ana Paula Taboada Sobral, Marcela Letícia Leal Gonçalves, Mayumi Oshiro Costa, Caroline Diniz Pagani Vieira Ribeiro, Mariana Moreira Machado, Abigail Malavasi, Elaine Marcílio Santos, Cinthya Cosme Gutierrez Duran, Sandra Kalil Bussadori

**Affiliations:** 1 Academic Master’s Program in Medicine in the Coastal Environment, Universidade Metropolitana de Santos (UNIMES), Santos 11045-002, Brazil; jusagretti@uol.com.br; 2 Postgraduate Program in Biophotonics Applied to Health Sciences, Universidade Nove de Julho (UNINOVE), São Paulo 01504-001, Brazil; amandahonorio@hotmail.com (A.R.H.M.); caroldvieirap@gmail.com (C.D.P.V.R.); cinthya.cgduran@gmail.com (C.C.G.D.); 3 Postgraduate Program in Health and Environment, Universidade Metropolitana de Santos (UNIMES), Santos 11045-002, Brazil; anapaula@taboada.com.br (A.P.T.S.); marcelalleal@hotmail.com (M.L.L.G.); elaine.marcilio@unimes.br (E.M.S.); 4 School of Dentistry, Universidade Metropolitana de Santos (UNIMES), Santos 11045-002, Brazil; 5 School of Psychology, Universidade Metropolitana de Santos (UNIMES), Santos 11045-002, Brazil; mayumioshiro@outlook.com; 6 School of Medicine, Universidade Metropolitana de Santos (UNIMES), Santos 11045-002, Brazil; mmachadomarianas@gmail.com; 7 Professional Master’s Program in Teaching Practices in Elementary Education, Universidade Metropolitana de Santos (UNIMES), Santos 11045-002, Brazil; abigail.malavasi@unimes.com

**Keywords:** halitosis, sleep quality, anxiety, depression, stress, university students

## Abstract

**Highlights:**

**Public health relevance—How does this work relate to a public health issue?**

- Halitosis is a common condition that may affect social interactions, psychological well-being, and quality of life among university students.
- Understanding the relationship between halitosis, sleep quality, anxiety, and stress contributes to a broader public health perspective on student health and well-being.

**Public health significance—Why is this work of significance to public health?**

- This study investigates the association between halitosis, psychological factors, and sleep quality in a population vulnerable to mental health challenges.
- The findings contribute to understanding the complex relationships among oral health, psychological factors, and sleep quality in university students.

**Public health implications—What are the key implications or messages for practitioners, policy makers and/or researchers in public health?**

- Objective assessment of oral malodor may contribute to a more accurate evaluation of halitosis in university populations.
- Further research is needed to clarify the relationships among oral health, sleep quality, and psychological well-being in university students and to inform future health promotion strategies.

**Abstract:**

Introduction: Halitosis is a common oral condition that may negatively affect social interactions, psychological well-being, and quality of life. However, evidence regarding its association with mental health and sleep quality remains inconsistent, particularly among university students. Aim: This cross-sectional study investigated the prevalence of objectively measured halitosis and its association with tongue coating, sleep quality, and symptoms of anxiety, depression, and stress among university students from the metropolitan region of Santos, Brazil. Methods: Sixty-two Brazilian university students enrolled in health-related undergraduate programs participated in the study; most were in the first academic year (61.3%) and resided in the municipality of Santos (59.7%). Halitosis was assessed using a portable breath detector, tongue coating was evaluated using the Coated Tongue Index, sleep quality was assessed using the Pittsburgh Sleep Quality Index (PSQI), and symptoms of depression, anxiety, and stress were measured using the Depression, Anxiety and Stress Scale (DASS-21). Results: Objectively measured halitosis was identified in 25 participants (40.3%; 95% CI: 29.0–52.7%). The analyses did not provide statistically significant evidence of associations between objectively measured halitosis and tongue coating, sleep quality, or symptoms of anxiety, depression, and stress. Significant positive correlations were identified between stress and sleep disturbance, anxiety and sleep disturbance, and depression and sleep latency, daytime dysfunction, and overall sleep quality. Tongue-cleaning habits were significantly associated with the presence of halitosis, whereas oral mucosal desquamation was less frequent among participants with halitosis. Conclusion: The study did not provide statistically significant evidence of associations between objectively measured halitosis and tongue coating, sleep quality, or psychological symptoms in this population. Significant positive correlations were identified between stress and sleep disturbance, anxiety and sleep disturbance, and depression and sleep latency, daytime dysfunction, and overall sleep quality. These findings highlight the importance of objectively assessing oral malodor in epidemiological studies.

## 1. Introduction

Halitosis is a common oral condition characterized by an unpleasant odor originating from the oral cavity that may negatively affect social interactions, psychological well-being, and quality of life [1,2,3]. Its psychosocial impact may be particularly relevant among young adults and university students, who are exposed to intense social interactions, academic demands, psychosocial stressors, and lifestyle changes [1,2,3,4]. The relationship between halitosis and psychological factors is complex, as clinically detectable oral malodor may contribute to psychological distress, whereas emotional factors may also influence the perception of breath odor [1,2,5,6].

The etiology of halitosis is multifactorial, although approximately 80–90% of cases originate within the oral cavity. Oral malodor is primarily associated with the microbial degradation of organic substrates and the production of volatile sulfur compounds (VSCs). Tongue coating, periodontal conditions, oral hygiene, salivary characteristics, microbial composition, and behavioral factors may contribute to the development and persistence of halitosis [2,7,8,9,10,11,12].

Growing evidence indicates that psychological factors may influence both the perception and manifestation of halitosis. Anxiety, depression, stress, and social anxiety disorder have been associated with self-perceived and clinically diagnosed halitosis [2,6,9,10,13]. Individuals reporting halitosis frequently experience embarrassment, reduced self-confidence, social avoidance, and concerns regarding interpersonal acceptance, reinforcing the complex relationship between oral conditions and psychological health [3,14,15]. Importantly, previous studies have suggested that psychological distress may exert a greater influence on the perception of oral malodor than on objectively measured halitosis, which may partly explain the inconsistent findings reported in the literature [1,2,9,10,14].

Previous studies conducted among university students in Bangladesh and Saudi Arabia reported a prevalence of self-perceived halitosis of approximately 56% and 55%, respectively [3,4]. However, these estimates should be interpreted with caution because self-perception may be influenced by sociocultural context, including norms related to interpersonal communication, personal hygiene, social acceptability, and willingness to report a potentially embarrassing condition. Psychological characteristics, individual awareness, and oral health behaviors may also affect the recognition and reporting of oral malodor. Consequently, self-perceived halitosis does not necessarily correspond to clinically detectable oral malodor [8,9,10].

Sleep quality is increasingly recognized as an important determinant of physical and psychological health [16,17]. University students are particularly susceptible to sleep disturbances due to academic workload, irregular routines, lifestyle changes, and psychosocial stressors [16,17,18,19]. Poor sleep quality has consistently been associated with anxiety, depression, stress, reduced quality of life, and adverse health outcomes [19,20,21,22,23,24,25]. Furthermore, insufficient sleep has been associated with self-reported halitosis in adolescents, suggesting a possible interaction between sleep-related factors and oral malodor [11]. Proposed biological mechanisms include reduced salivary flow, alterations in the oral microbiota, mouth breathing, and behavioral changes associated with sleep deprivation, all of which may contribute to the production of volatile sulfur compounds [11,26,27].

Although increasing attention has been devoted to the relationships among oral health, mental health, and sleep quality, evidence regarding the interplay between objectively measured halitosis, psychological symptoms, and sleep disturbances remains limited, particularly among university students. Most available studies have investigated self-perceived halitosis or examined these conditions independently, resulting in heterogeneous and, at times, contradictory findings [3,4,14,15]. Moreover, relatively few investigations have simultaneously evaluated objectively diagnosed halitosis, tongue coating, sleep quality, and psychological symptoms within the same population. This gap limits the understanding of the complex interactions between oral and psychological health and highlights the need for multidisciplinary research using objective diagnostic methods.

Therefore, the present study aimed to investigate the prevalence of objectively measured halitosis and its association with tongue coating, sleep quality, and symptoms of anxiety, depression, and stress among university students from the metropolitan region of Santos, Brazil. We hypothesized that objectively measured halitosis would be associated with greater tongue coating, poorer sleep quality, and higher levels of anxiety, depression, and stress.

## 2. Materials and Methods

### 2.1. Study Design and Ethical Aspects

This descriptive, quantitative, observational, cross-sectional study was conducted using a non-probabilistic convenience sample of university students from the metropolitan region of Santos, São Paulo, Brazil. The study was approved by the Human Research Ethics Committee of Universidade Metropolitana de Santos (UNIMES) on 5 December 2023 (CAAE: 74442423.0.0000.5509; Approval No. 6.556.493) and conducted in accordance with the ethical principles of the Declaration of Helsinki and its subsequent amendments. The study design and reporting followed the Strengthening the Reporting of Observational Studies in Epidemiology (STROBE) guidelines. The completed STROBE checklist is provided as Supplementary Material.

### 2.2. Study Objectives and Hypotheses

The objective of this study was to determine the prevalence of objectively measured halitosis and to investigate its associations with tongue coating, sleep quality, and symptoms of depression, anxiety, and stress among university students from the metropolitan region of Santos, Brazil. We hypothesized that participants with objectively measured halitosis would present greater tongue coating, poorer sleep quality, and higher levels of depression, anxiety, and stress than participants without halitosis.

### 2.3. Participants

The study included university students aged 18 years or older enrolled in health-related undergraduate programs at the Universidade Metropolitana de Santos (UNIMES), located in the metropolitan region of Santos, São Paulo, Brazil. Students were recruited from different semesters of the courses in Medicine, Psychology, Nursing, Physiotherapy, and Pharmacy programs.

Students were excluded if they lived outside the metropolitan region of Santos, were younger than 18 years, had dentofacial anomalies, were undergoing orthodontic or orthopedic treatment, were receiving cancer treatment, had self-reported systemic conditions potentially associated with oral malodor, such as gastrointestinal, renal, or liver disease, or had used antibiotics in the month prior to data collection.

### 2.4. Data Collection Procedures

Data collection was conducted between 15 March 2024 and 30 June 2024 during previously scheduled sessions held at the participating university facilities. Assessments were conducted in classrooms for students enrolled in the Medicine, Psychology, Nursing, and Physiotherapy programs and in the clinical training facilities used by Pharmacy students.

**Figure 1:**
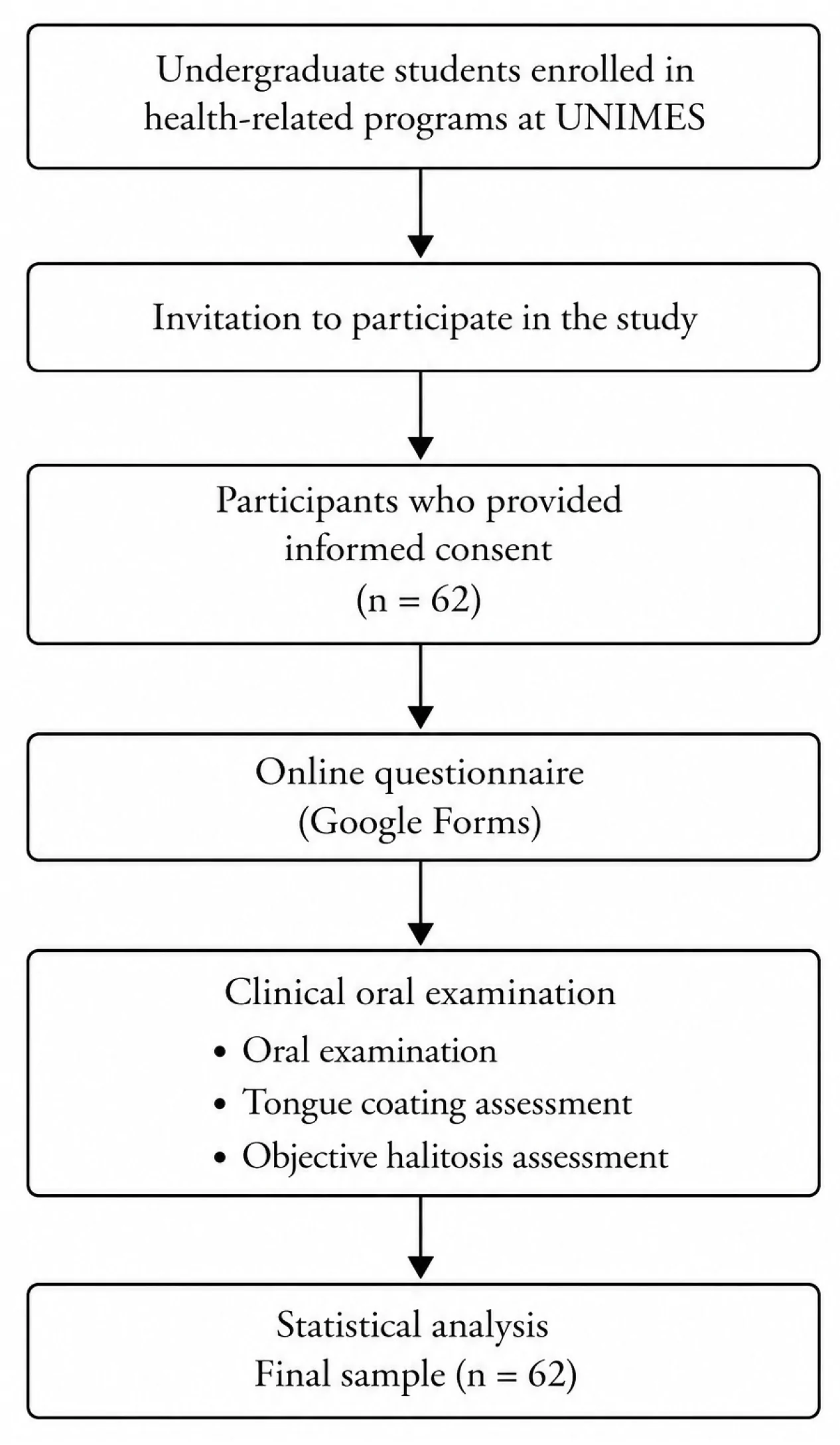
Flow diagram of participant recruitment and study procedures.

Participants received standardized pre-assessment instructions regarding tongue-coating assessment and breath evaluation 24 and 48 h before the scheduled examination, with the aim of standardizing conditions before the clinical measurements. On the day of data collection, all participants received detailed information regarding the study objectives and procedures and provided electronic informed consent before participation. Participants subsequently completed an online questionnaire using their own mobile devices. The questionnaire was administered in Brazilian Portuguese using Google Forms and completed electronically by the participants. Questionnaires with incomplete responses were excluded from the final analysis. The estimated completion time was approximately 15 min. Immediately after completing the questionnaire, clinical assessment of tongue coating and breath analysis were performed by the same examiner, who had undergone practical training and procedural standardization in the study assessment protocol, including operation of the portable breath detector and assessment of tongue coating. No formal intra-examiner reproducibility coefficient was calculated. The participant recruitment and study procedures are summarized in Figure 1.

### 2.5. Assessment Measures

The questionnaire consisted of validated instruments (PSQI and DASS-21), together with a structured questionnaire developed by the research team to collect demographic characteristics, oral health habits, and general health information relevant to the study objectives. Sleep quality was evaluated using the Pittsburgh Sleep Quality Index (PSQI), originally developed by Buysse et al. and culturally adapted and validated for the Brazilian population by Bertolazi et al. [28,29]. The PSQI evaluates sleep quality during the previous month through 19 self-administered items grouped into seven components: subjective sleep quality, sleep latency, sleep duration, habitual sleep efficiency, sleep disturbances, use of sleep medication, and daytime dysfunction. Component scores range from 0 to 3, resulting in a global score ranging from 0 to 21, with higher scores indicating poorer sleep quality. Global scores > 5 were considered indicative of poor sleep quality.

Symptoms of depression, anxiety, and stress were assessed using the Brazilian Portuguese version of the Depression, Anxiety and Stress Scale (DASS-21) [30,31,32]. The instrument comprises 21 items equally distributed across three domains: depression, anxiety, and stress. Responses are recorded using a four-point Likert scale ranging from 0 (“Did not apply to me at all”) to 3 (“Applied to me very much or most of the time”). Scores for each domain were calculated by summing the corresponding items, with higher scores indicating greater symptom severity.

Information regarding oral and general health was collected through a structured questionnaire addressing oral hygiene practices, tongue-cleaning habits, xerostomia, morning dry mouth, gingival bleeding, self-perceived halitosis, dental caries, gingival alterations, oral mucosal desquamation, smoking habits, mouth breathing, gastrointestinal complaints, and other self-reported health conditions potentially associated with oral malodor.

Tongue coating was assessed using the Coated Tongue Index proposed by Shimizu et al. [33]. The dorsal surface of the tongue was divided into nine anatomical areas. Each area received a score of 0 (absence of coating), 1 (coating with visible lingual papillae), or 2 (coating completely covering the papillae). The final index was calculated by summing the scores, dividing the total by 18, and multiplying the result by 100, yielding a percentage ranging from 0% to 100%.

Halitosis was assessed using a portable KKCare (KKcare, China) breath detector according to the manufacturer’s recommendations. The device provides a semiquantitative assessment of breath odor using a five-point scale. No independent validation against a reference method was performed in the present sample; therefore, the device was used as a screening tool rather than as a reference diagnostic method. Participants were instructed to exhale continuously into the device for approximately 5 s. Breath odor was classified as 0 (good), 1 (normal), 2 (not very good), 3 (bad), or 4 (very bad). For statistical analyses, scores ≥ 2 were considered indicative of halitosis.

### 2.6. Statistical Analysis

Data were analyzed using IBM SPSS Statistics, version 28.0 (SPSS Inc., Chicago, IL, USA). The Shapiro–Wilk test was used to assess the normality of continuous variables. Descriptive statistics were calculated, with continuous variables expressed as means and standard deviations and categorical variables expressed as absolute and relative frequencies.

Associations between categorical variables were analyzed using the chi-square test or Fisher’s exact test, when appropriate. For the statistically significant categorical associations identified in the exploratory analyses, crude odds ratios (ORs) with corresponding 95% confidence intervals were calculated from 2 × 2 contingency tables to provide an estimate of the magnitude and precision of the observed associations. Spearman’s correlation coefficient was used to assess correlations between halitosis levels, tongue-coating index, DASS-21 domains, and PSQI components, as this test is suitable for ordinal or non-normally distributed variables. To quantify the precision of the estimates, 95% confidence intervals (95% CIs) for Spearman’s rho were estimated using a bias-corrected and accelerated (BCa) nonparametric bootstrap procedure with 50,000 paired resamples. The 95% CI for the prevalence of halitosis was estimated using the Wilson score method. Due to the convenience sampling strategy and the exploratory nature of the study, an a priori sample size calculation was not performed. Post hoc statistical power was calculated for the statistically significant correlations to provide additional information regarding the robustness of the observed associations. Nevertheless, the relatively small sample size may have limited the ability to detect weaker associations and should be considered when interpreting the findings. The analyses were exploratory. Multivariable modeling was not performed because only 25 participants met the criterion for objectively measured halitosis and several potentially relevant covariates had low frequencies or sparse cells. Under these conditions, simultaneous adjustment for multiple covariates would result in a low number of outcome events relative to the number of parameters estimated, increasing the risk of overfitting, unstable regression coefficients, inflated standard errors, and separation. Restricting the model to a small subset of covariates selected post hoc was also avoided because this could introduce data-driven model-selection bias. Therefore, the analyses were intentionally restricted to exploratory bivariate associations. A *p*-value < 0.05 was considered statistically significant.

## 3. Results

### 3.1. Participant Characteristics

Table 1 summarizes the demographic and academic characteristics of the 62 university students included in the study. Medicine students represented the largest proportion of the sample (35.5%), followed by students from the remaining health-related programs. Most participants were in the first academic year (61.3%) and resided in the municipality of Santos (59.7%). Stomach disorders were self-reported by 16.1% of the participants, whereas 12.9% reported current smoking.

### 3.2. Oral Health Characteristics and Halitosis

The mean tongue-coating index was 46.4 ± 27.7, and the prevalence of objectively measured halitosis was 40.3% (25/62; 95% CI: 29.0–52.7%). Regarding oral hygiene practices, 71.0% of the participants reported using dental floss, 51.6% used mouthwash, and 46.8% performed tongue cleaning. Participants reported brushing their teeth an average of 1.7 ± 0.6 times per day. Additional oral health characteristics and factors potentially associated with halitosis are presented in Table 2.

### 3.3. Mental Health and Sleep Quality

The assessment of psychological symptoms using the DASS-21 revealed that stress had the highest mean score (9.2 ± 3.7), followed by anxiety and depression. Sleep quality, assessed using the PSQI, showed a mean global score of 7.8 ± 2.7. Among the PSQI domains, sleep latency presented the highest mean score (1.7 ± 0.7). Detailed results for the DASS-21 and PSQI domains are presented in Table 3.

### 3.4. Correlations Between Halitosis, Tongue Coating, Mental Health, and Sleep Quality

Spearman’s correlation analysis did not provide statistically significant evidence of correlations between halitosis levels and the tongue-coating index, psychological symptoms, or sleep quality indicators (Table 4).

Significant correlations were identified between mental health indicators and sleep quality parameters (Table 5). The stress domain was positively correlated with sleep disturbance (rho = 0.418; 95% CI: 0.212 to 0.600; *p* = 0.001; 1 − β = 0.93). The depression domain was positively correlated with sleep latency (rho = 0.271; 95% CI: 0.013 to 0.492; *p* = 0.033; 1 − β = 0.57), daytime dysfunction (rho = 0.287; 95% CI: 0.031 to 0.504; *p* = 0.024; 1 − β = 0.62), and the global PSQI score (rho = 0.298; 95% CI: 0.060 to 0.509; *p* = 0.018; 1 − β = 0.66). The anxiety domain was positively correlated with sleep disturbance (rho = 0.366; 95% CI: 0.129 to 0.561; *p* = 0.003; 1 − β = 0.84). For the remaining comparisons, the analyses did not provide statistically significant evidence of correlations between the mental health domains and sleep quality components.

### 3.5. Associations Between Halitosis and Oral Health Variables

Associations between the presence of halitosis and oral health variables are presented in Table 6. A significant association was observed between halitosis and tongue-cleaning habits (*p* = 0.009). Participants with halitosis had higher odds of reporting tongue cleaning than those without halitosis (crude OR = 4.43; 95% CI: 1.49–13.12). Oral mucosal desquamation was less frequent among participants with halitosis (*p* = 0.038; crude OR = 0.11; 95% CI: 0.01–0.94).

## 4. Discussion

The principal finding and main contribution of the present study is that objectively measured halitosis did not reproduce the pattern of associations with psychological symptoms and sleep quality frequently reported in studies based on self-perceived halitosis. Objectively measured halitosis was identified in 40.3% of the university students, but the analyses did not provide statistically significant evidence of associations with anxiety, depression, stress, sleep quality, or tongue coating. In contrast, tongue-cleaning habits and oral mucosal desquamation were associated with halitosis. These findings emphasize the importance of distinguishing self-perceived oral malodor from objectively detected halitosis when interpreting epidemiological associations.

Previous studies among university students have reported self-perceived halitosis prevalences of approximately 55–56% [3,4], whereas a study involving dentistry students reported self-perceived halitosis in 33.8% and objectively assessed oral malodor in 47.5% of participants [34]. Self-perceived halitosis may be influenced not only by oral conditions but also by psychological, cultural, behavioral, and social factors [2,9,10,14,15]. Consequently, associations identified in studies based on self-report may partly reflect factors related to the perception of breath odor rather than objectively detectable oral malodor itself. This distinction offers a plausible explanation for why the present findings differ from several previous reports.

The use of an objective assessment represents an important methodological feature of the present study. However, the portable sulfide monitor should be regarded as a screening tool rather than a reference diagnostic method. Although such devices provide a rapid and non-invasive assessment of oral malodor, they primarily detect total volatile sulfur compounds and do not differentiate individual gases or their oral and extraoral origins [7,8,35]. Moreover, the device was not independently validated against a reference method in the present sample, and its semiquantitative nature may have introduced some degree of measurement imprecision, potentially reducing the ability to detect weaker associations. These methodological characteristics should be considered when comparing the present findings with studies using organoleptic assessment or gas chromatography.

The analyses did not provide statistically significant evidence of associations between objectively measured halitosis and anxiety, depression, stress, or sleep quality. This contrasts with reports linking halitosis to psychological distress and social anxiety [1,2,10,13], although much of this evidence concerns self-perceived halitosis, which may be influenced by emotional factors, self-esteem, body image, and social anxiety [2,3,4,5,6,7,8,9,10]. The distinction between self-perceived and objectively measured halitosis may therefore partly explain the different pattern observed in the present study. The limited sample size may have also provided insufficient statistical power to detect weak associations, while the relative homogeneity of students enrolled predominantly in health-related programs may have restricted the range and variability of psychological measures.

In contrast, significant correlations were identified among the psychological and sleep variables themselves: stress was positively correlated with sleep disturbance, anxiety with sleep disturbance, and depression with sleep latency, daytime dysfunction, and overall sleep quality. These correlations were weak to moderate and should be interpreted cautiously given the exploratory design and limited sample size. Their direction is consistent with the established relationship between sleep and mental health in university populations [19,20,21,22,23,24,25,36]. In a recent IJERPH study involving 397 university students, Priede et al. [25] similarly found that poorer sleep quality was moderately correlated with depression, anxiety, and stress and proposed that such multidimensional evidence could inform student mental-well-being support programs. Taken together, these findings support a broader interpretation of student health in which sleep, emotional distress, health behaviors, and oral-health concerns may interact, even when objectively measured halitosis itself is not statistically associated with psychological symptoms. Biological mechanisms linking psychological stress and sleep disturbance may involve increased physiological arousal and alterations in neuroendocrine and inflammatory pathways [17,18,19,20,22,26].

Regarding oral conditions, the analysis did not provide statistically significant evidence of a correlation between the tongue-coating index and objectively measured halitosis. Volatile sulfur compound production depends not only on the amount of tongue coating but also on its microbial composition, substrate availability, salivary characteristics, oral hygiene, and other individual clinical factors [5,7,8,12,27,37]. Similar variability has been reported in specific populations, including mouth-breathing children [38]. Thus, tongue coating should be interpreted as one component of the multifactorial etiology of halitosis rather than as an isolated determinant.

An unexpected finding was the significant association between tongue-cleaning habits and halitosis. Because of the cross-sectional design, this association should not be interpreted as evidence that tongue cleaning increases the risk of oral malodor. A plausible alternative explanation is reverse directionality: individuals who are already aware of or concerned about their breath may be more likely to adopt tongue cleaning in an attempt to control it.

Oral mucosal desquamation was less frequent among participants with halitosis. Although previous reports have suggested that epithelial desquamation may contribute to the availability of organic substrates for bacterial degradation [39], the mechanisms underlying this finding remain uncertain. Salivary flow, mucosal integrity, oral microbiota, and local inflammatory conditions may influence both epithelial turnover and volatile sulfur compound production [40]. Therefore, this association should be regarded as exploratory and hypothesis-generating rather than as evidence of a protective effect.

Several limitations of the present study should be acknowledged. First, the cross-sectional design does not permit determination of the temporal sequence of the observed associations or establishment of causal relationships. Second, the study used a non-probabilistic convenience sample recruited from a single university, predominantly comprising students enrolled in health-related programs. This relatively homogeneous sample may limit the generalizability of the findings and may also have reduced the variability of some investigated characteristics. In addition, self-selection bias cannot be excluded, as students who agreed to participate may have differed systematically from those who did not participate.

Halitosis was assessed at a single time point using a portable sulfide monitor. Although this approach provided an objective, rapid, and standardized screening assessment, the device was not independently validated against a reference method in the present sample, measures total volatile sulfur compounds, and does not differentiate individual gases or their oral and extraoral sources [7,8,35]. A comprehensive periodontal examination was not performed, and potentially relevant factors such as salivary flow and composition, dietary intake, medication use beyond the exclusion criterion for recent antibiotic therapy, and the microbiological composition and metabolic activity of the tongue biofilm were not comprehensively assessed or controlled.

Furthermore, several oral and general health variables, including xerostomia, gastrointestinal complaints, dental caries, gingival alterations, and other health conditions, were based on participant self-report rather than comprehensive clinical confirmation. Misclassification or reporting bias therefore cannot be excluded. Because multivariable modeling was not considered statistically reliable given the small number of participants with halitosis and the sparse distribution of several candidate covariates, the reported associations remain unadjusted, and residual confounding cannot be excluded. Finally, multiple statistical comparisons were performed without adjustment for multiplicity, increasing the possibility of false-positive findings (type I error). The results should therefore be interpreted as exploratory and hypothesis-generating and require confirmation in larger, longitudinal studies with more comprehensive clinical assessments.

Overall, the findings reinforce the importance of distinguishing objectively detected oral malodor from self-perceived halitosis when interpreting associations with psychological and behavioral factors. From a broader student well-being perspective, the observed interrelationships among stress, anxiety, depression, and sleep suggest that these domains should not be considered or addressed in isolation. Recent multidimensional research has similarly emphasized the use of combined mental-health, sleep, and behavioral evidence to inform student support programs [25].

Although coping strategies and quality of life were not directly assessed in the present study, evidence from university populations indicates that stress intensity, coping patterns, psychological well-being, and quality of life are interconnected [41]. A recent scoping review also highlighted the diversity of coping strategies used by university students and the importance of developing student-informed and context-sensitive approaches to stress prevention and management [42]. Furthermore, systematic reviews of resilience and mindfulness-based interventions suggest that institutional programs may contribute to reductions in stress, anxiety, and depressive symptoms and may support some dimensions of student well-being, although the effectiveness varies according to the intervention and outcome assessed [43,44].

From a proactive public-health perspective, universities may therefore benefit from integrated and non-stigmatizing support pathways combining stress-management and adaptive-coping resources, sleep-health education, psychosocial support, and access to oral-health assessment. Future longitudinal and mixed-methods studies should jointly evaluate coping strategies, quality of life, sleep, psychological symptoms, and objectively measured oral conditions to clarify their temporal relationships and identify modifiable targets for student support.

## 5. Conclusions

Objectively measured halitosis was identified in 25 of the 62 university students (40.3%), and the analyses did not provide statistically significant evidence of associations with tongue coating, psychological symptoms, or sleep quality. Tongue-cleaning habits and oral mucosal desquamation were associated with halitosis, whereas psychological symptoms were correlated with specific sleep-quality components. Given the cross-sectional design, limited sample size, and multiple statistical comparisons, these findings should be interpreted as exploratory and require confirmation in larger longitudinal studies.

## Figures and Tables

**Table 1:** Sociodemographic, academic, and self-reported health characteristics of the university students (*n* = 62; n [%]).

	n (%)
**Demographic characteristics**	
*Course*	
Nursing	11 (17.7)
Pharmacy	11 (17.7)
Physiotherapy	7 (11.3)
Medicine	22 (35.5)
Psychology	11 (17.7)
*Years of course*	
1 year	38 (61.3)
2 to 3 years	12 (19.4)
≥4 years	12 (19.4)
**Municipality of residence**	
Bertioga	1 (1.6)
Cubatão	2 (3.2)
Mongaguá	3 (4.8)
Praia Grande	9 (14.5)
Santos	37 (59.7)
São Vicente	10 (16.1)
**Risk factors (% yes)**	
Heart problems	0 (0.0)
Stomach problems	10 (16.1)
Intestinal problems	1 (1.6)
Mouth breathing	6 (9.7)
Diabetes	2 (3.2)
Use of illicit substances	6 (9.7)
Smoker	8 (12.9)

The data are presented as absolute frequency (n) and percentage (%).

**Table 2:** Oral health conditions, oral hygiene practices, and halitosis prevalence in the study sample (*n* = 62; mean ± SD, %, or *n* [%], as applicable).

Variable	Value
**Continuous variables, mean ± SD**	
Coated tongue index	46.4 ± 27.7
Toothbrushing frequency, number per day	1.7 ± 0.6
**Categorical variables, %** Presence of halitosis Brand of toothpaste	25 (40.3)
Colgate	62.9
Oral B	25.8
Sorriso	8.1
Use of mouthwash	51.6
Use of dental floss	71.0
Habit of cleaning tongue	46.8
Gingival bleeding	21.0
Dry mouth	14.5
Dry mouth upon waking	22.6
Grinding of teeth	11.3
Perception of bad breath	33.9
Presence of caries	27.4
Gingival alterations	21.0
Desquamation of oral mucosa	17.7

Continuous variables are presented as mean ± standard deviation (SD). Categorical variables are presented as relative frequencies (%), except for the prevalence of halitosis, which is presented as n (%).

**Table 3:** DASS-21 domain scores and PSQI component scores in the study sample (*n* = 62; mean ± SD).

	Mean and Standard Deviation
**Depression, Anxiety and Stress Scale**	
Depression domain	5.5 ± 4.1
Stress domain	9.2 ± 3.7
Anxiety domain	6.1 ± 4.1
**Pittsburgh Sleep Quality Index**	
Subjective sleep quality	1.5 ± 0.6
Sleep latency	1.7 ± 0.7
Sleep duration	1.1 ± 0.8
Habitual sleep efficiency	0.4 ± 0.8
Sleep disturbance	1.2 ± 0.5
Use of sleep medications	0.3 ± 0.9
Daytime dysfunction	1.6 ± 0.7
Overall sleep quality score	7.8 ± 2.7

Data are presented as mean ± standard deviation.

**Table 4:** Correlations of objectively measured halitosis levels with tongue coating, DASS-21 domains, and PSQI components (*n* = 62; Spearman’s rho [95% CI; *p*]).

Variables	Halitosis Level rho (95% CI; *p*)
Tongue coating index	0.194 (−0.054 to 0.414; *p* = 0.130)
**Depression, Anxiety and Stress Scale**	
Depression domain	−0.019 (−0.271 to 0.230; *p* = 0.884)
Stress domain	0.017 (−0.239 to 0.268; *p* = 0.895)
Anxiety domain	−0.049 (−0.300 to 0.202; *p* = 0.707)
**Pittsburgh Sleep Quality Index**	
Subjective sleep quality	−0.118 (−0.369 to 0.157; *p* = 0.362)
Sleep latency	−0.033 (−0.252 to 0.183; *p* = 0.801)
Sleep duration	0.235 (−0.018 to 0.452; *p* = 0.066)
Habitual sleep efficiency	0.233 (−0.020 to 0.459; *p* = 0.068)
Sleep disturbance	−0.022 (−0.262 to 0.232; *p* = 0.865)
Use of sleep medications	0.106 (−0.197 to 0.382; *p* = 0.412)
Daytime dysfunction	0.120 (−0.128 to 0.354; *p* = 0.353)
Overall sleep quality score	0.094 (−0.163 to 0.338; *p* = 0.468)

Values are presented as Spearman’s correlation coefficients (rho), 95% bias-corrected and accelerated (BCa) bootstrap confidence intervals, and *p*-values.

**Table 5:** Correlations between DASS-21 domains and PSQI components in the study sample (n = 62; Spearman’s rho [95% CI; *p*]).

	Depression, Anxiety and Stress Scale rho (95% CI; *p*)		
Variables	Depression Domain	Stress Domain	Anxiety Domain
**Pittsburgh Sleep Quality Index**			
Subjective sleep quality	−0.044 (−0.304 to 0.233; *p* = 0.735)	−0.002 (−0.281 to 0.277; *p* = 0.988)	−0.029 (−0.292 to 0.237; *p* = 0.820)
Sleep latency	0.271 (0.013 to 0.492; *p* = 0.033) *	0.063 (−0.203 to 0.315; *p* = 0.626)	0.171 (−0.094 to 0.414; *p* = 0.184)
Sleep duration	0.054 (−0.214 to 0.310; *p* = 0.679)	−0.097 (−0.362 to 0.185; *p* = 0.454)	0.015 (−0.249 to 0.275; *p* = 0.910)
Habitual sleep efficiency	0.042 (−0.191 to 0.277; *p* = 0.749)	−0.070 (−0.345 to 0.221; *p* = 0.588)	0.049 (−0.217 to 0.308; *p* = 0.705)
Sleep disturbance	0.247 (−0.024 to 0.483; *p* = 0.053)	0.418 (0.212 to 0.600; *p* = 0.001) *	0.366 (0.129 to 0.561; *p* = 0.003) *
Use of sleep medications	0.229 (−0.025 to 0.445; *p* = 0.073)	0.077 (−0.247 to 0.329; *p* = 0.553)	0.155 (−0.195 to 0.398; *p* = 0.230)
Daytime dysfunction	0.287 (0.031 to 0.504; *p* = 0.024) *	0.135 (−0.135 to 0.392; *p* = 0.296)	0.204 (−0.074 to 0.453; *p* = 0.112)
Overall sleep quality score	0.298 (0.060 to 0.509; *p* = 0.018) *	0.039 (−0.231 to 0.311; *p* = 0.761)	0.190 (−0.060 to 0.419; *p* = 0.138)

Values are presented as Spearman’s correlation coefficients (rho), 95% bias-corrected and accelerated (BCa) bootstrap confidence intervals, and *p*-values. * *p* < 0.05.

**Table 6:** Associations of objectively measured halitosis with oral health, behavioral, sleep, and general health variables (*n* = 62; n [%]).

Variable	No Halitosis (n = 37), n (%)	Halitosis (n = 25), n (%)	*p*-Value
Use of mouthwash	19 (51.4)	13 (52.0)	1.000
Use of dental floss	23 (62.2)	21 (84.0)	0.088
Tongue-cleaning habit	12 (32.4)	17 (68.0)	0.009
Gingival bleeding	8 (21.6)	5 (20.0)	1.000
Dry mouth	5 (13.5)	4 (16.0)	1.000
Dry mouth upon waking	10 (27.0)	4 (16.0)	0.367
Grinding the teeth	4 (10.8)	3 (12.0)	1.000
Perception of bad breath	13 (35.1)	8 (32.0)	1.000
Presence of caries	13 (35.1)	6 (24.0)	0.410
Gingival alterations	10 (27.0)	3 (12.0)	0.210
Desquamation of oral mucosa	10 (27.0)	1 (4.0)	0.038
Poor sleep quality (PSQI > 5)	31 (83.8)	21 (84.0)	1.000
Stomach problems	7 (18.9)	3 (12.0)	0.726
Intestinal problems	1 (2.7)	0 (0.0)	1.000
Mouth breathing	5 (13.5)	1 (4.0)	0.387
Diabetes	2 (5.4)	0 (0.0)	0.511
Use of illicit substances	4 (10.8)	2 (8.0)	1.000
Smoker	7 (18.9)	1 (4.0)	0.128

Data are presented as absolute frequency and percentage, n (%), of participants reporting each characteristic within the groups without halitosis and with halitosis. *p*-values were obtained using Fisher’s exact test.

## Data Availability

The data presented in this study are available from the corresponding author upon reasonable request. The data are not publicly available due to privacy and ethical restrictions.

## Supplementary Materials

The following supporting information can be downloaded at: https://www.mdpi.com/article/10.3390/ijerph23081043/s1, STROBE Statement—checklist of items that should be included in reports of cross-sectional studies.

## Abbreviations

The following abbreviations are used in this manuscript:

DASS-21	Depression, Anxiety and Stress Scale–21
PSQI	Pittsburgh Sleep Quality Index
VSC	Volatile Sulfur Compound
STROBE	Strengthening the Reporting of Observational Studies in Epidemiology

## References

[1] He M., Lu H., Cao J., Zhang Y., Wong M.C.M., Fan J., Ye W. (2020). Psychological characteristics of Chinese patients with genuine halitosis. Oral Dis..

[2] Zaitsu T., Ueno M., Shinada K., Wright F.A., Kawaguchi Y. (2011). Social anxiety disorder in genuine halitosis patients. Health Qual. Life Outcomes.

[3] Dey A., Khan M.A.S., Eva F.N., Islam T., Hawlader M.D.H. (2024). Self-perceived halitosis and associated factors among university students in Dhaka, Bangladesh. BMC Oral Health.

[4] Mezied M.S., Al Bouri D., Al Omani A., Al Ramadhan G., Al Bootie S., Barakat A., Koppolu P. (2023). A cross-sectional study on self-perceived halitosis among undergraduate university students in Riyadh, Saudi Arabia. J. Pharm. Bioallied Sci..

[5] Aylıkcı B.U., Colak H. (2013). Halitosis: From diagnosis to management. J. Nat. Sci. Biol. Med..

[6] Calil C.M., Marcondes F.K. (2006). Influence of anxiety on the production of oral volatile sulfur compounds. Life Sci..

[7] Porter S.R., Scully C. (2006). Oral malodour (halitosis). BMJ.

[8] Bicak D.A. (2018). A current approach to halitosis and oral malodor: A mini review. Open Dent. J..

[9] Vali A., Roohafza H., Keshteli A.H., Afghari P., Shirani M.J., Afshar H., Savabi O., Adibi P. (2015). Relationship between subjective halitosis and psychological factors. Int. Dent. J..

[10] Eli I., Baht R., Koriat H., Rosenberg M. (2001). Self-perception of breath odor. J. Am. Dent. Assoc..

[11] Do K.Y. (2020). Relationship between insufficient sleep and bad breath in Korean adolescent population. Int. J. Environ. Res. Public Health.

[12] Zhang H., Zhang T., Fu X., Qin H., Chen X., Zeng X. (2025). Prevalence and associated factors of halitosis among university students in Guangxi, Southern China: A cross-sectional study. Oral Health Prev. Dent..

[13] Kursun S., Acar B., Atakan C., Oztas B., Paksoy C.S. (2014). Relationship between genuine and pseudohalitosis and social anxiety disorder. J. Oral Rehabil..

[14] Briceag R., Caraiane A., Raftu G., Horhat R.M., Bogdan I., Fericean R.M., Shaaban L., Popa M., Bumbu B.A., Bratu M.L. (2023). Emotional and social impact of halitosis on adolescents and young adults: A systematic review. Medicina.

[15] Cassiano L.S., Abdullahi F., Leite F.R.M., Lopez R., Peres M.A., Nascimento G.G. (2021). The association between halitosis and oral-health-related quality of life: A systematic review and meta-analysis. J. Clin. Periodontol..

[16] Buboltz W.C., Brown F., Soper B. (2001). Sleep habits and patterns of college students: A preliminary study. J. Am. Coll. Health.

[17] Al-Khani A.M., Sarhandi M.I., Zaghloul M.S., Ewid M., Saquib N. (2019). A cross-sectional survey on sleep quality, mental health, and academic performance among medical students in Saudi Arabia. BMC Res. Notes.

[18] Ghrouz A.K., Noohu M.M., Manzar M.D., Spence D.W., BaHammam A.S., Pandi-Perumal S.R. (2019). Physical activity and sleep quality in relation to mental health among college students. Sleep Breath..

[19] Wang F., Bíró É. (2021). Determinants of sleep quality in college students: A literature review. Explore.

[20] Baglioni C., Nanovska S., Regen W., Spiegelhalder K., Feige B., Nissen C., Reynolds C.F., Riemann D. (2016). Sleep and mental disorders: A meta-analysis of polysomnographic research. Psychol. Bull..

[21] Ford D.E., Kamerow D.B. (1989). Epidemiologic study of sleep disturbances and psychiatric disorders: An opportunity for prevention?. JAMA.

[22] Alvaro P.K., Roberts R.M., Harris J.K. (2013). A systematic review assessing bidirectionality between sleep disturbances, anxiety, and depression. Sleep.

[23] Dülger H., Ayaz-Alkaya S. (2025). Prevalence of stress, anxiety, depression, and sleep quality among young adults in Turkiye: A cross-sectional study. J. Eval. Clin. Pract..

[24] Gao R., Wang H., Liu S., Wang X., Song S., Wang Y. (2024). Study on anxiety, depression, and sleep conditions and their interrelations among vocational college students during the COVID-19 pandemic management normalization. Front. Public Health.

[25] Priede L., Beitane I., Beitane L. (2025). A study of relationships between mental well-being, sleep quality, eating behavior, and BMI: A cross-sectional study among university students. Int. J. Environ. Res. Public Health.

[26] Majde J.A., Krueger J.M. (2005). Links between the innate immune system and sleep. J. Allergy Clin. Immunol..

[27] Lei F., Hu X. (2025). Oral health and sleep disorders: A systematic review and meta-analysis. Biomed. Rep..

[28] Bertolazi A.N., Fagondes S.C., Hoff L.S., Dartora E.G., da Silva Miozzo I.C., de Barba M.E.F., Menna Barreto S.S. (2011). Validation of the Brazilian Portuguese version of the Pittsburgh Sleep Quality Index. Sleep Med..

[29] Buysse D.J., Reynolds C.F., Monk T.H., Berman S.R., Kupfer D.J. (1989). The Pittsburgh Sleep Quality Index: A new instrument for psychiatric practice and research. Psychiatry Res..

[30] Lovibond S.H., Lovibond P.F. (1995). Manual for the Depression Anxiety Stress Scales.

[31] Martins B.G., Silva W.R., Maroco J., Campos J.A.D.B. (2019). Escala de Depressão, Ansiedade e Estresse: Propriedades psicométricas e prevalência das afetividades. J. Bras. Psiquiatr..

[32] Vignola R.C.B., Tucci A.M. (2014). Adaptation and validation of the Depression, Anxiety and Stress Scale (DASS) to Brazilian Portuguese. J. Affect. Disord..

[33] Shimizu T., Ueda T., Sakurai K. (2007). New method for evaluation of tongue-coating status. J. Oral Rehabil..

[34] Schemel-Suárez M., Chimenos-Küstner E., Estrugo-Devesa A., Jané-Salas E., López-López J. (2017). Halitosis assessment and changes in volatile sulfur compounds after chewing gum: A study performed on dentistry students. J. Evid. Based Dent. Pract..

[35] Guedes C.C., Bussadori S.K., Garcia A.C.M., Motta L.J., Gomes A.O., Weber R., Amancio O.M.S. (2020). Accuracy of a portable breath meter test for the detection of halitosis in children and adolescents. Clinics.

[36] Demenech L.M., Oliveira A.T., Neiva-Silva L., Dumith S.C. (2021). Prevalence of anxiety, depression and suicidal behaviors among Brazilian undergraduate students: A systematic review and meta-analysis. J. Affect. Disord..

[37] Jazzar A., AlDehlawi H., Farag A., Alhamed S., Akeel S., Mair Y., Flemban K., Alqassab H., Aljohani K. (2024). Clinical parameters in patients with halitosis: A cross-sectional study. Front. Dent. Med..

[38] Bruno L.H., Mandetta A.R.H., Sobral A.P.T., Leal Gonçalves M.L., Santos E.M., Fossati A.L., Gallo J.M.A.S., Motta P.B., Deana A.M., Horliana A.C.R.T. (2024). Assessment of photodynamic therapy with annatto and LED for the treatment of halitosis in mouth-breathing children: Randomized controlled clinical trial. PLoS ONE.

[39] Bastos Y.V.P., Menezes P.A.F., Gomes J.L.R., Silva L.T.C., Peixoto F.B. (2017). Diagnóstico diferencial de ardência bucal: Relato de caso. Rev. Acad. Bras. Odontol..

[40] Kolbe A.C., Silva F.L. (2017). Uso da isotretinoína no tratamento da acne e sua relação com a halitose. Rev. Ciênc. Méd. Biol..

[41] Kupcewicz E., Grochans E., Kadučáková H., Mikla M., Jóźwik M. (2020). Analysis of the relationship between stress intensity and coping strategy and the quality of life of nursing students in Poland, Spain and Slovakia. Int. J. Environ. Res. Public Health.

[42] Waterhouse P., Samra R. (2026). University students’ coping strategies to manage stress: A scoping review. Educ. Rev..

[43] Abulfaraj G.G., Upsher R., Zavos H.M.S., Dommett E.J. (2024). The impact of resilience interventions on university students’ mental health and well-being: A systematic review. Educ. Sci..

[44] Pan Y., Li F., Liang H., Shen X., Bing Z., Cheng L., Dong Y. (2024). Effectiveness of mindfulness-based stress reduction on mental health and psychological quality of life among university students: A GRADE-assessed systematic review. Evid. Based Complement. Altern. Med..

